# Atypical Presentation Resembling Acute Leukoencephalopathy With Restricted Diffusion in Staphylococcus aureus Meningoencephalitis

**DOI:** 10.7759/cureus.55517

**Published:** 2024-03-04

**Authors:** Jai Ranjan, Arvinder Wander, Navdeep Kaur, Bhawna Sharma, Kamla Kant, Akriti Aggarwal

**Affiliations:** 1 Microbiology, All India Institute of Medical Sciences, Bathinda, Bathinda, IND; 2 Pediatrics, All India Institute of Medical Sciences, Bathinda, Bathinda, IND; 3 Radiodiagnosis, All India Institute of Medical Sciences, Bathinda, Bathinda, IND

**Keywords:** mrsa, alerd, leukoencephalopathy, staphylococcus aureus, meningoencephalitis

## Abstract

Meningoencephalitis refers to inflammation of the brain and meninges. It can be caused by various organisms, such as *Neisseria meningitidis*, *Streptococcus pneumoniae*, and so on. *Staphylococcus aureus* causing meningoencephalitis is relatively rare. It is mainly encountered in patients who have undergone surgeries in the past. Acute leukoencephalopathy with restricted diffusion (ALERD) is a type of encephalopathy that can involve both white and grey matter of the brain, and it has a characteristic “bright tree appearance” on MRI. It can be because of various infectious etiologies or caused by various toxins. Neurological sequelae are observed in about two out of three cases. Here, we describe a case of *S. aureus* meningoencephalitis with ALERD, which has been seldom reported. More awareness about this is required among primary care physicians for timely diagnosis and management to prevent any complications.

## Introduction

Inflammation of the brain and meninges is referred to as meningoencephalitis. It is an acute condition that requires prompt medical attention. Patients presenting with seizures, altered sensorium, and fever should be evaluated for meningoencephalitis or central nervous system (CNS) infections. It can lead to mortality in about 11-25% of cases [1]. Hence, rapid diagnosis and treatment are required to prevent long-term neurological sequelae [2]. It can be caused by viruses such as herpes simplex virus; bacteria such as *Mycobacterium tuberculosis*, *Streptococcus pneumoniae*, *Hemophilus influenzae*, *Neisseria meningitidis*, and so on; fungal pathogens such as *Cryptococcus neoformans*; and parasitic infections [2]. *Staphylococcus aureus *is rarely implicated in bacterial meningitis. CNS infections due to *S. aureus *usually occur as a complication of neurological procedures [3] or injury or skull base surgeries [4].

Acute injury to the brain can result in neurological symptoms such as seizures, loss of consciousness, and so on, which is referred to as acute encephalopathy. Bacterial infections or meningitis can rarely lead to acute encephalopathy. Recently, acute encephalopathy with biphasic seizures with restricted diffusion (AESD) has been described because of infectious etiology. It is characterized by fever with seizures initially, followed by a stage of apparent clinical improvement, which is again succeeded by episodes of seizures. MRI done during the second stage of seizures exhibits areas of restricted diffusion in the brain [5]. The term acute leukoencephalopathy with restricted diffusion (ALERD) is used for children presenting with seizures, encephalopathy symptoms, and restricted diffusion on MRI at presentation itself. It can be because of various infections and does not have a biphasic clinical picture [6]. Long-term neurological sequelae can be observed in patients presenting with ALERD. Thus, immediate management of the condition is of utmost importance.

Here, we present the case of a child with *S. aureus* meningoencephalitis with ALERD, which is an unusual etiology.

## Case presentation

**Figure 1:**
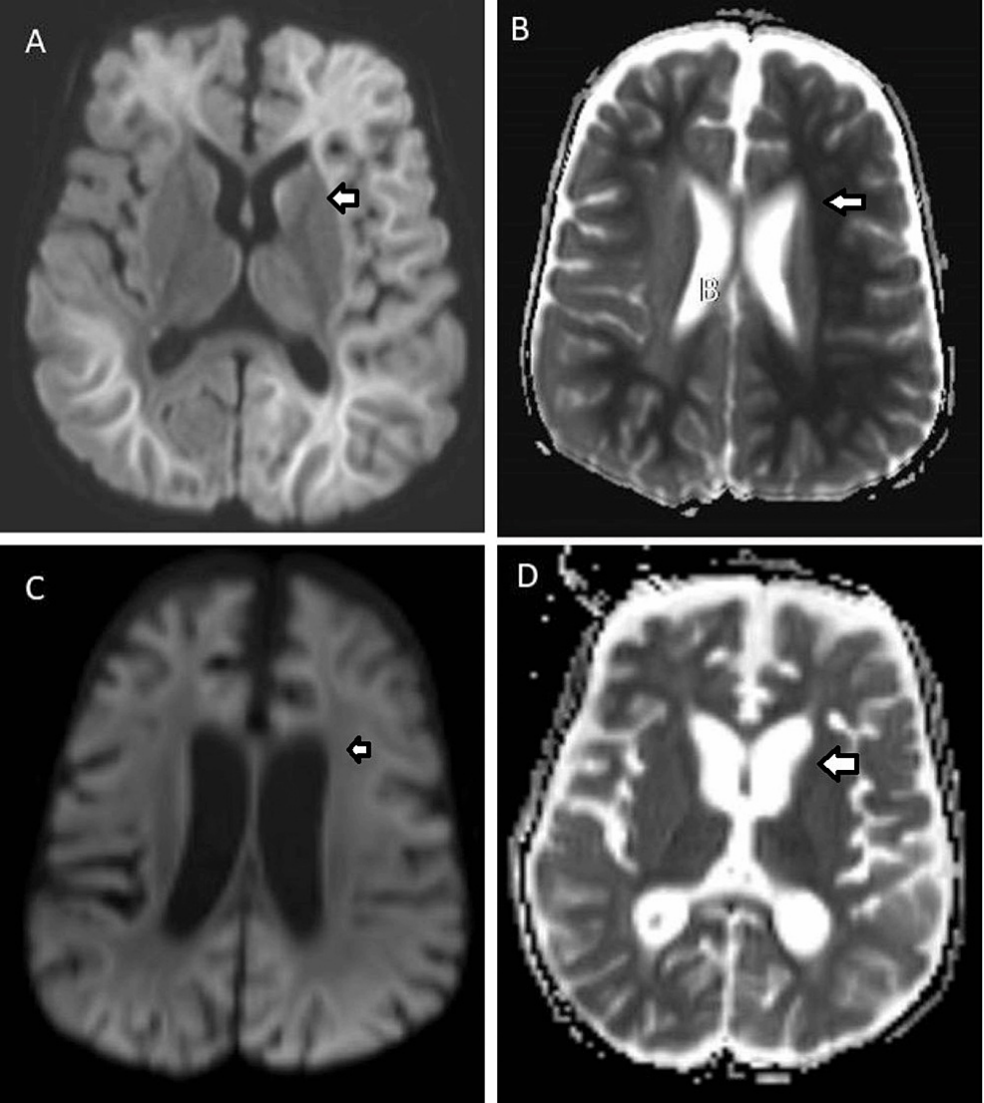
MRIs of the brain of the patient (arrows showing areas of interest) A. Diffusion-weighted MRI shows diffuse and confluent areas of diffusion restriction involving the bilateral cerebral white matter at the time of first presentation (arrow showing areas of interest) B. Apparent diffusion coefficient (ADC) map corresponding to the image in A shows a dark signal in the involved white matter, suggestive of true diffusion restriction (arrow showing areas of interest) C. Diffusion-weighted MRI two weeks after the initial presentation shows normalization of signal with no restricted diffusion in the white matter (arrow showing areas of interest) D. ADC map corresponding to C shows a normal signal in the white matter with no diffusion restriction (arrow showing areas of interest)

A one-year-old girl presented with a history of fever, altered sensorium, loose stools, and seizures. On examination, the patient was lethargic; both pupils were sluggishly reactive, and neck rigidity was present. Injection of ceftriaxone, vancomycin, and acyclovir were started, considering the possibility of acute encephalitis. An MRI of the brain revealed confluent true diffusion restriction involving the periventricular white matter in a symmetric fashion, which suggests an acute ALERD-like picture (Figure 1).

Routine investigations at presentation showed neutrophilia and elevated liver enzymes (Table 1).

**Table 1:** Test parameters at presentation

Test parameters	Value	Reference range
Hemoglobin	4.5 g/dL	11.0-16.0 g/dL
White blood cells	17.68 x 10^3^/µL	4.00-11.00 x 10^3^/µL
Monocyte percentage	4.0%	3.0-12.0%
Neutrophil percentage	78.4%	50.0-70%
Basophil percentage	0.2%	0.0-1.0%
Eosinophil percentage	0.1%	0.5-5.0%
Platelet	373 x 10^3^/µL	150-450 x 10^3^/µL
C-reactive protein (quantitative)	22.4 mg/L	<6 mg/L
Chloride	100.20 mEq/L	98-107 mEq/L
Potassium	3.97 mEq/L	3.5-5.1 mEq/L
Sodium	128.20 mEq/L	136-145 mEq/L
Albumin	3.0 g/dL	3.5-5.2 g/dL
Alkaline phosphatase	144 IU/L	53-128 IU/L
Direct bilirubin	0.17 mg/dL	0.0-0.30 mg/dL
Total bilirubin	0.56 mg/dL	0.0-2.00 mg/dL
Globulin	1.60 g/dL	2.5-3.5 g/dL
Aspartate transaminase	1,968 U/L	0.0-35 U/L
Alanine transaminase	2,048 U/L	0.0-45 U/L
Total protein	4.6 g/dL	6.4-8.3 g/dL
Creatinine	0.31 mg/dL	0.6-1.10 mg/dL
Urea	21.3 mg/dL	15-45 mg/dL
CSF glucose	41.8 mg/dL	40-70 mg/dL
CSF proteins	58.4 mg/dL	<50 mg/dL

**Figure 2:**
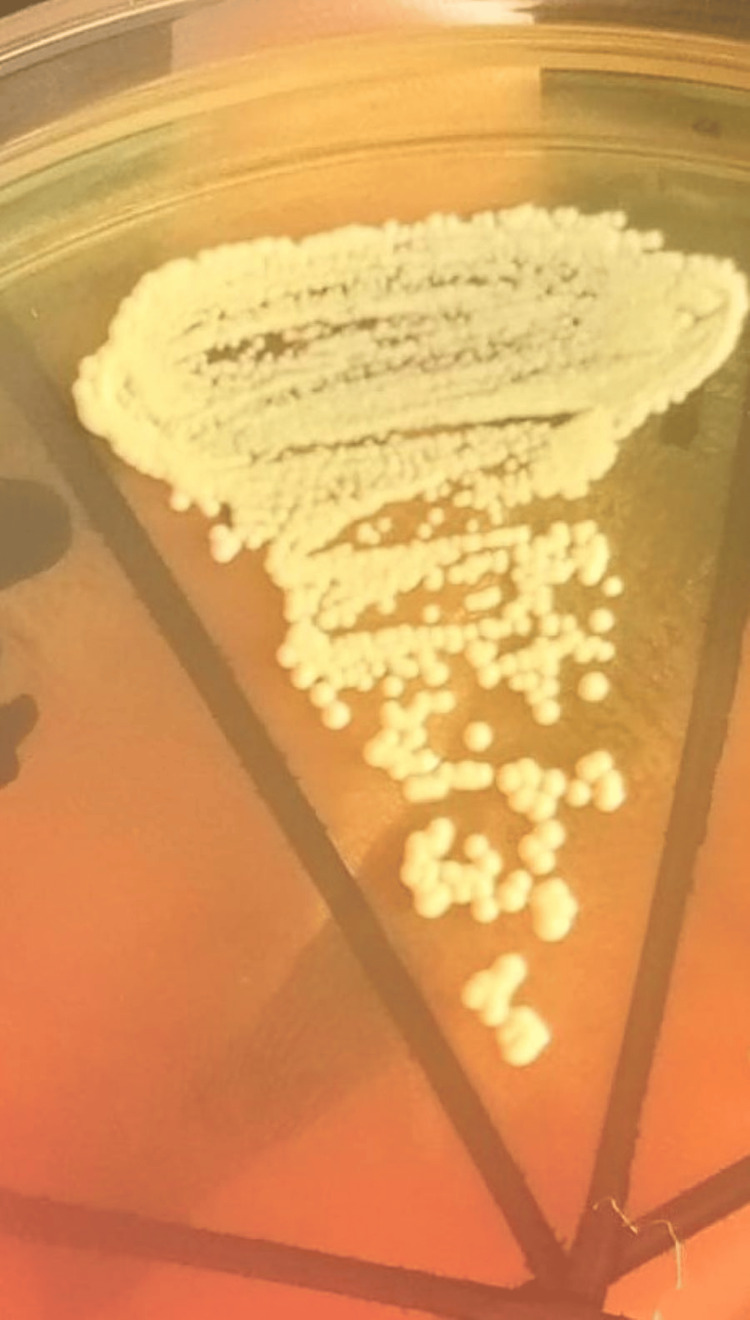
Yellow-colored mannitol fermenting S. aureus colonies on mannitol salt agar

Blood was sent for dengue IgG/IgM, NS1, and malaria antigen test, and all were negative. Laboratory investigations of CSF revealed a neutrophil count of 429/cumm. The CSF protein and glucose levels were 58.4 mg/dL and 41.8 mg/dL (against a blood sugar level of 130 mg/dL), respectively. The CSF was sent for culture in microbiology. A Gram’s stain of CSF showed pus cells with gram-positive cocci in clusters. On culture, beta-hemolytic colonies on blood agar and yellow colonies on mannitol salt agar (Figure 2) were seen. It was identified as methicillin-resistant S. aureus (MRSA) by Vitek-2 compact (bioMérieux, Marcy l'Etoile, France). The MRSA was susceptible to linezolid and vancomycin. Additionally, the CSF was negative for herpes simplex virus.

The patient was continued on injection vancomycin for 21 days, which resulted in clinical improvement. Routine investigations after treatment showed normal levels of liver enzymes, and repeat CSF culture was sterile. Follow-up MRI done after two weeks of initial presentation showed complete normalization of diffusion restriction in white matter (Figure 1). The child was discharged in stable condition with no further episodes of seizures.

## Discussion

According to Global Burden of Disease, 2016, meningitis incidence rose from 2.50 million in 1990 to 2.82 million in 2016. About 1.2 million people are annually affected with bacterial meningitis [7]. The incidence of meningitis and meningoencephalitis was 12.1/100,000 patient-years in a study by Gofrit et al. [8]. The incidence of bacterial meningitis in Northern India ranged from 3% to 21.8% [7]. Hasbun et al. in their study, which included children up to 17 years old, found the incidence of meningitis or encephalitis to be highest in children less than one year of age (45%) [9]. Thus, if meningoencephalitis affects children, it can result in significant mortality and morbidity.

Predominantly, viruses are implicated as causative agents in meningitis or encephalitis. Only about 13% of infections are caused by bacterial pathogens [9]. Common bacterial pathogens causing meningitis are *S. pneumoniae*, *H. influenzae*, and *N. meningitidis* [7]. *S. aureus* is rarely implicated in bacterial meningitis. Meningoencephalitis due to *S. aureus *is uncommon [10,11]. Group B *Streptococcus*, *S. pneumoniae*, *Escherichia coli*, *H. influenzae*, and so on, are common causes of meningitis in neonates and children [12]. *S. aureus* as a cause of meningoencephalitis is predominantly observed after surgical procedures, shunt placements [3], and in immunocompromised individuals. In our case, the child presented with fever and altered sensorium and was found to have an ALERD-like picture on radiological examination.

CSF sent for routine investigations showed the CSF glucose to blood glucose ratio to be 0.32, which is suggestive of bacterial meningitis [13]. Liver enzymes are usually elevated in diffuse ALERD, as in our patient [5,14]. The patient was treated with vancomycin. Improvement in clinical condition and normalization of radiological findings were observed after the completion of therapy for MRSA meningoencephalitis. 

This is a rare case report wherein ALERD can be attributed to MRSA meningoencephalitis. *S. aureus* meningoencephalitis is seldom reported, and it is usually observed in patients undergoing any neurological procedures. Our patient did not have any history of previous surgeries, thus making this a unique case. Contamination of CSF culture is unlikely, as pus cells were observed on the Gram’s stain of the CSF sample, which points to a likely infectious pathology. Recovery and improvement of the radiological picture after completion of MRSA therapy also substantiates MRSA as a causative etiology of ALERD in our case.

ALERD refers to an acute encephalopathy with restricted diffusion, such as the picture in both the grey and white matter of the brain on an MRI scan. It can be because of infectious or toxin-related mechanisms. Recently, even snake bites among children have also been found to cause it [15]. It is an encephalopathy syndrome that can be a result of infections because of adenovirus, coxsackievirus, *E. coli*, and so on. [5]. In a study by Lawrence et al., dengue hemorrhagic fever was the most common cause of ALERD [6]. Ischemia and brain infarction can also result in a similar picture [5]. Patients with ALERD present with fever, altered sensorium, and encephalopathy. Seizures lasting up to 30 minutes, which could be generalized or partial, can be observed. About two-thirds of the patients can have neurological complications [5].

Two types of ALERD are described in the literature. One, which spares the central region of the brain, is of a milder variety. Diffuse ALERD involves all the regions of the brain, is severe, and can also result in coma [5,16]. Moreover, it is predominantly associated with raised liver enzymes, metabolic acidosis, and hyperglycemia. Hypercytokinemia, excitotoxic injury, and neuronal death are proposed to be the probable pathogeneses behind the clinical picture [17]. Diffusion-weighted image (DWI) and ADC maps during MRI scans are required to diagnose ALERD, with “bright tree appearance” being the characteristic feature of DWI in MRI scans of the brain [5].

Since in most cases, there are residual neurological issues such as cognitive impairment or delay [6] and speech problems [14], evaluation of all cases of ALERD is of vital importance. Matsuo et al., in their study, evaluated dextromethorphan and cyclosporine A for ALERD cases and found them to be effective in most patients [18]. Further studies and trials are required to evaluate the treatment options for it.

## Conclusions

In our case, the child was found to have ALERD on radiological examination after an episode of meningoencephalitis because of *S. aureus*. ALERD in meningoencephalitis because of *S. aureus* is relatively uncommon. As such a condition can lead to neurological sequelae, it is of vital importance for physicians to investigate, treat, and diagnose patients presenting with symptoms of seizures, altered sensorium, and so on. More research is required on the treatment options for ALERD to prevent neurological complications in patients.
